# Discordance in glycemic categories and regression to normality at baseline in 10,000 people in a Type 2 diabetes prevention trial

**DOI:** 10.1038/s41598-018-24662-y

**Published:** 2018-04-19

**Authors:** Mike Sampson, Tim Elwell-Sutton, Max O. Bachmann, Allan Clark, Ketan K. Dhatariya, Clare Ferns, Amanda Howe, W. Garry John, Gerry Rayman, Leyla Swafe, Jeremy Turner, Melanie Pascale

**Affiliations:** 1 Norfolk Diabetes Prevention Study (NDPS), Elsie Bertram Diabetes Centre, Norfolk and Norwich University Hospital NHS Trust, Norwich, UK; 2 Norwich Medical School (NMS), University of East Anglia (UEA), Norwich, UK; 3 Department Clinical Biochemistry, Norfolk and Norwich University Hospital NHS Trust, Norwich, UK; 4 Department of Diabetes and Endocrinology, Ipswich General Hospital, Ipswich, UK

## Abstract

The world diabetes population quadrupled between 1980 and 2014 to 422 million and the enormous impact of Type 2 diabetes is recognised by the recent creation of national Type 2 diabetes prevention programmes. There is uncertainty about how to correctly risk stratify people for entry into prevention programmes, how combinations of multiple ‘at high risk’ glycemic categories predict outcome, and how the large recently defined ‘at risk’ population based on an elevated glycosylated haemoglobin (HbA1c) should be managed. We identified all 141,973 people at highest risk of diabetes in our population, and screened 10,000 of these with paired fasting plasma glucose and HbA1c for randomisation into a very large Type 2 diabetes prevention trial. Baseline discordance rate between highest risk categories was 45.6%, and 21.3–37.0% of highest risk glycaemic categories regressed to normality between paired baseline measurements (median 40 days apart). Accurate risk stratification using both fasting plasma glucose and HbA1c data, the use of paired baseline data, and awareness of diagnostic imprecision at diagnostic thresholds would avoid substantial overestimation of the true risk of Type 2 diabetes and the potential benefits (or otherwise) of intervention, in high risk subjects entering prevention trials and programmes.

## Introduction

The world diabetes population quadrupled between 1980 and 2014 to 422 million, with an estimated global prevalence in 2014 of 9.0% (95% credible interval 7.2–11.1%) in men and 7.9% (6.4–9.7%) in women^1,2^. The enormous impact of Type 2 diabetes is recognised by the calls for international focus on this issue^1,3^ and the need for more effective diabetes prevention strategies^4,5^.

The early Type 2 diabetes prevention trials showed a significant impact in reducing the risk of progression to Type 2 diabetes over 3 years in intensively managed research populations, largely with impaired glucose tolerance (IGT)^6,7^. Smaller, and more pragmatic studies, then tested the translatability of similar interventions^8^. Meta – analysis of these later studies suggested the probability of metabolic benefit in the short term, though with little evidence for immediate impact in Type 2 diabetes prevention, particularly in the less well studied (but very prevalent) high risk groups defined by HbA1c criteria^8–10^. The recent shift from glucose based to HbA1c based diagnostic criteria for diabetes has created new substantial populations with non - diabetic hyperglycemia (NDH; HbA1c ≥6.0%–<6.5%, ≥42–<48 mmol/mol), identified as at ‘high risk of Type 2 diabetes’, but where clinical trial evidence of diabetes prevention benefit from lifestyle intervention is very modest^8–11^. However, in 2015 the evidence base was seen as strong enough in the UK to mandate a national diabetes prevention programme in England, with entry to the short term intervention programme triggered largely by an NDH HbA1c diagnosis (HbA1c ≥6.0%–<6.5%, ≥42–<48 mmol/mol)^12,13^. Some concerns have been expressed in the UK about this ambitious national programme and this approach^14,15^. There is also a lack of current real world data on the prevalence, characteristics, and accessibility of the various at risk glycemic categories in a UK primary care population which makes planning more difficult^12,13^. The detailed, strongly evidence based, and multiagency USA national effort to develop an effective national programme for Type 2 diabetes prevention in the USA has also been described very recently^16^.

Entry to Type 2 diabetes prevention programmes in the UK is now commonly triggered by a single data point of an elevated HbA1c ≥6.0%–<6.5%, (≥42–<48 mmol/mol) (NDH). This approach largely ignores the rich longitudinal epidemiological data describing both HbA1c and fasting plasma glucose as continuous predictor variables for incident Type 2 diabetes risk, ignores the discordance between these glycemic categories, and ignores the substantial added value of combining both HbA1c and fasting plasma glucose in the accurate risk prediction for incident Type 2 diabetes^17–22^. This issue is important, as an estimated 10.7% of the adult population in England now have NDH^23^, and national guidance is that these patients (perhaps 4 million people in England) should now have targeted diet and lifestyle advice to reduce the risk of Type 2 diabetes 11. There may be capacity problems in delivering this.

To deliver effective intervention and diabetes prevention programmes, we need to understand the heterogeneity in the at risk populations entering prevention trials and programmes, and the opportunities for more accurate risk classification, risk stratification, in this and similar populations. The aim of this study is to describe the baseline characteristics and heterogeneity of the first 10,000 participants at ‘high risk of Type 2 diabetes’ in a UK population screened with paired fasting plasma glucose and HbA1c for randomisation into the largest current UK diabetes prevention trial.

## Participants and Methods

The Norfolk Diabetes Prevention Study (NDPS; www.norfolkdiabetespreventionstudy.nhs.uk) is a 7 year programme funded by the UK National Institute of Health Research (NIHR RP-PG–0109–10013) and the full programme has been described elsewhere^24^. NDPS commenced in 2011 and reports in 2018. The programme tests the efficacy of an intensive 46 month lifestyle intervention in reducing the risk of transition to Type 2 diabetes for people with various ‘prediabetes’ or ‘non - diabetic hyperglycemia’ combinations^11,17,18,23^. The programme takes into account cost and workload pressures, and uses volunteer lay trainers with Type 2 diabetes to support the intervention, and randomises subjects with an elevated HbA1c (≥6%–<6.5%; ≥42–<48 mmol/mol) or impaired fasting glucose (IFG; fasting plasma glucose ≥5.6–<7.0 mmol/L) or combinations of these categories, but does not undertake oral glucose tolerance tests as a primary screening test to detect people with impaired glucose tolerance (IGT)^11,17,18^.

The classification and terminology for people at high risk of Type 2 diabetes based on a glucose or HbA1c that are elevated, but not into the diabetes diagnostic range, is complex and includes overlapping categories of prediabetes, impaired fasting glucose (IFG), impaired glucose tolerance (IGT), impaired glucose regulation (IGR), or non - diabetic hyperglycemia (NDH)^11,17^. In this paper, we restrict the term non - diabetic hyperglycemia (NDH) to participants with HbA1c ≥6%–<6.5% (≥42–<48 mmol/mol) and impaired fasting glucose (IFG) to participants with fasting plasma glucose of ≥6.1–<7.0 mmol/L or ≥5.6–<7.0 mmol/L depending on the classification criteria used^11,17,18^.

The programme identifies people at high risk of these categories through existing general practice (primary care) databases, and screens invited participants with paired baseline fasting plasma glucose and HbA1c to identify those suitable for randomisation. We contacted 194 general practices in Norfolk, Suffolk and North East Essex in England and by 1^st^ March 2016, 135 practices were active collaborators, with a primary care population of 1.8 million. The NDPS used the existing NHS primary care electronic health record (EHR) software such as SystmOne or EMIS in each practice. All people without known diabetes in these practices were contacted if EHR data suggested they (**a**) were age ≥50 years with BMI ≥ 30 kg/m^2^ or (**b**) age ≥50 years and BMI ≥ 25 kg/m^2^ if there was also a recorded first degree family history of Type 2 diabetes, a history of coronary artery disease, or gestational diabetes or **c**) had any previous record of impaired fasting glucose (IFG) or impaired glucose tolerance (IGT) or a fasting plasma glucose of ≥6.1–<7.0 mmol/L or (**d**) any previous record of HbA1c ≥6.0%–<6.5% (≥42–<48 mmol/mol) and a fasting glucose ≥5.6–<6.1 mmol/L. The mean prevalence of these categories on GP databases were 11% (a and b combined), 1.7% (c), and 2.7% (d) respectively. We contacted 141,973 people, inviting them to participate and 12,778 (9%) registered for participation, with the 10,000^th^ consecutive participant completed on 1.3.16. After written informed consent, participants underwent venesection for fasting plasma glucose and HbA1c, and biometric and clinical data collection. Participants found to have any elevated fasting glucose or HbA1c in a randomisable category on initial testing were informed they had an abnormal (elevated) result and were invited to return for a repeat fasting plasma glucose and HbA1c measurement under the same conditions and at the same site, which was undertaken a median 40 days (interquartile range 27–69 days) later. Participants with a normal fasting plasma glucose (<5.6 mmol/l) or HbA1c <42 mmol/mol were not invited to return for a repeat sample. Randomisation into trial only occurred if paired baseline tests were concordant for glycemic category. In the early years of this programme (2011–2013), before changes to the new diagnostic criteria for diabetes based on HbA1c^11,24^ and changes in randomisation criteria in this programme, not all participants with an NDH level HbA1c, or a fasting plasma glucose ≥5.6–<6.1 mmol/L, had a repeat test as these categories (or combinations of categories) were not randomised into trials at the time. Fasting plasma glucose was measured by a hexokinase/G-6-PDH method (Architect c8000: Abbott Diagnostics, Maidenhead, UK). HbA1c was measured using Affinity high performance liquid chromatography (Hb9210: Menarini Diagnostics Ltd., Wokingham, UK). Weight, body mass index (BMI), body fat mass, visceral fat, and body fat percentage was measured using a Tanita body fat composition analyser (TANITA - Hoogoorddreef, 1101 BE, Amsterdam,The Netherlands. Model BC-420 MA). Data are shown as n (%) or mean and one standard deviation (SD). Probability for trend across all categories was calculated using linear regression, logistic regression, and Spearman correlation tests for continuous, binary and categorical variables respectively suing Stata software (Stata 14.1/SE. StataCorp. 2015. Stata Statistical Software,StataCorp LP). All subjects gave written informed consent to research participation after ethical review and approval from the National Research Ethics Service (NRES), Essex 1 Research Ethics Committee (10/H0301/55; 13.1.2011). All methods were performed in accordance with NRES permissions and after research governance approval form the sponsor organisation (Norfolk and Norwich University Hospital NHS Trust). In line with pre specified NDPS analysis plans, we undertook analysis of the first consecutive 10,000 patients screened.

## Results

### Clinical characteristics

The baseline characteristics of the first 10,000 screened participants are shown stratified by glycemic category (Tables 1 and 2), and by age and body mass index (Table 3).

**Table 1:** Baseline characteristics stratified by HbA1c category for 10,000 participants at high risk of Type 2 diabetes screened for randomisation into the Norfolk Diabetes Prevention Study.

	HbA1c % (mmol/mol)							
	<6% (<42)		≥6%–<6.5% (≥42–<48)		≥6.5% (≥48)		p^a^	p trend^b^
N (%)	7342	(73.4)	2172	(21.7)	486	(4.9)		
Age (years)	61.3	(9.7)	65.2	(9.6)	64.5	(9.9)	<0.001	<0.001
Sex, n (%)								
*Female*	3870	(52.9)	1055	(48.6)	209	(43.0)		
*Male*	3448	(47.1)	1117	(51.4)	277	(57.0)	<0.001	<0.001
Ethnicity								
*White British*	6871	(93.6)	2059	(94.8)	438	(90.1)		
*White other*	182	(2.5)	48	(2.2)	19	(3.9)		
*Asian*	38	(0.5)	22	(1.0)	6	(1.2)		
*Other*	57	(0.8)	16	(0.7)	9	(1.9)		
*Unknown*	194	(2.6)	27	(1.2)	14	(2.9)	<0.001	0.78
HbA1c (%)	5.6	(0.25)	6.1	(0.14)	7.0	(0.69)	<0.001	<0.001
HbA1c (mmol/mol)	37.4	(2.7)	43.7	(1.5)	52.8	(7.5)	<0.001	<0.001
Fasting plasma glucose (mmol/L)	5.1	(0.5)	5.7	(0.6)	6.8	(1.5)	<0.001	<0.001
Body mass index (BMI; kg/m^2^)	30.5	(6.9)	31.2	(5.8)	32.6	(6.4)	<0.001	<0.001
BMI Categories (n; %)								
*Low (*<*18.5)*	12	(0.2)	2	(0.1)	0	(0.0)		
*Normal (18.5 − 24.9)*	1122	(15.3)	253	(11.7)	48	(9.9)		
*Overweight (25–29.9)*	2113	(28.8)	685	(31.7)	125	(25.9)		
*Obese (30–39.9)*	3711	(50.7)	1071	(49.5)	252	(52.2)		
*Very obese* (≥*40)*	368	(5.0)	153	(7.1)	58	(12.0)	<0.001	<0.001
Percentage body fat (%)	36.4	(9.1)	37.1	(8.7)	38.3	(8.8)	<0.001	<0.001
Visceral fat (kg),	13.7	(4.6)	15.2	(4.8)	16.4	(5.4)	<0.001	<0.001
Waist circumference (cm)	103.3	(13.6)	106.4	(13.4)	110.9	(14.0)	<0.001	<0.001
Body fat mass (kg)	32.5	(11.9)	33.7	(11.9)	36.6	(13.4)	<0.001	<0.001
Smoking status, n (%)								
*Current smoker*	418	(5.8)	170	(8.0)	39	(8.1)		
*Ex-smoker*	3142	(43.3)	996	(46.8)	219	(45.5)		
*Never smoked*	3688	(50.9)	962	(45.2)	223	(46.4)	<0.001	<0.001

^a^p-value comparing the ‘normal’ <6% (<42 mmol/mol) group with the NDH group (HbA1c ≥6%–<6.5%, ≥42–<48 mmol/mol)

p-values from chi-squared test or Fisher exact test for small numbers.

^b^p-for trend calculated using linear regression for continuous variables, logistic regression for binary variables, Spearman correlation test for categorical variables. All data shown as n (%) or mean (1 standard deviation).

**Table 2:** Baseline characteristics stratified by fasting plasma glucose category for 10,000 participants at high risk of Type 2 diabetes, screened for randomisation into the Norfolk Diabetes Prevention Study.

	Fasting plasma glucose (mmol/L)									
	<5.6		5.6–6.0		6.1–6.9		≥7.0		p^a^	p trend^b^
n (%)	7106	(71.1)	1684	(16.8)	967	(9.7)	243	(2.4)		
Age (years)	61.5	(9.8)	64.2	(9.5)	64.7	(9.7)	62.7	(10.1)	0.20	<0.001
**Sex (n; %)**										
*Female*	3982	(56.2)	686	(40.8)	372	(38.6)	94	(38.7)		
*Male*	3107	(43.8)	994	(59.2)	592	(61.4)	149	(61.3)	0.20	<0.001
**Ethnicity**										
*White British*	6665	(93.8)	1572	(93.3)	903	(93.4)	228	(93.8)		
*White other*	172	(2.4)	45	(2.7)	28	(2.9)	4	(1.6)		
*Asian*	40	(0.6)	13	(0.8)	11	(1.1)	2	(0.8)		
*Other*	53	(0.7)	15	(0.9)	9	(0.9)	5	(2.1)		
*Unknown*	176	(2.5)	39	(2.3)	16	(1.7)	4	(1.6)	0.67	0.52
HbA1c (%)	5.6	(0.32)	5.9	(0.36)	6.1	(0.41)	7.0	(1.02)	<0.00	<0.001
HbA1c (mmol/mol)	38.1	(3.5)	41	(3.9)	43.7	(4.5)	53.5	(11.2)	<0.001	<0.001
Fasting glucose (mmol/L)	5	(0.4)	5.8	(0.1)	6.4	(0.2)	8	(1.4)	<0.001	<0.001
BMI; (kg/m^2^)	30.7	(7.0)	30.8	(5.6)	31	(5.8)	32.5	(5.9)	0.35	0.49
**BMI Categories n (%)**										
*Low (*<*18.5)*	13	(0.2)	1	(0.1)	0	(0.0)	0	(0.0)		
*Normal (18.5–24.9)*	1058	(14.9)	220	(13.1)	126	(13.1)	19	(7.8)		
*Overweight (25–29.9)*	1951	(27.5)	566	(33.7)	337	(35.0)	69	(28.4)		
*Obese(30–39.9)*	3692	(52.1)	787	(46.8)	428	(44.4)	127	(52.3)		
*Very obese*(≥*40)*	371	(5.2)	108	(6.4)	72	(7.5)	28	(11.5)	0.65	0.78
Percentage body fat (%)	36.9	(9.1)	35.7	(8.8)	35.8	(9.0)	37.7	(8.8)	0.85	<0.001
Visceral fat (kg)	13.7	(4.6)	15.1	(4.7)	15.5	(4.9)	16.4	(5.5)	0.10	<0.001
Waist circumference (cm)	103.4	(13.7)	105.9	(13.1)	106.8	(14.1)	110.2	(13.7)	0.12	<0.001
Body fat mass (kg)	32.8	(11.9)	32.6	(11.7)	33.2	(12.7)	36.4	(13.3)	0.23	0.50
**Smoking status, n (%)**										
*Current smoker*	453	(6.5)	93	(5.6)	56	(5.9)	25	(10.4)		
*Ex-smoker*	2993	(42.7)	794	(48.1)	467	(49.3)	103	(42.7)		
*Never smoked*	3571	(50.9)	765	(46.3)	424	(44.8)	113	(46.9)	0.74	<0.001

^a^p-value comparing the ≥5.6–<6.1 mmol/L fasting plasma glucose group with the ≥6.1–6.9 mmol/L group. p -values from chi-squared test or Fisher exact test for small numbers.

^b^p-for trend calculated using linear regression for continuous variables, logistic regression for binary variables, Spearman correlation test for categorical variables.

All data shown as n (%) or mean (1 standard deviation).

**Table 3:** Prevalence (%) and numbers of participants with impaired fasting glucose and/or non*–*diabetic hyperglycemia in a high risk population of 10,000*.

BMI (kg/m^2^)									
Age band (yrs)		<20	20<25	25–<30	30–<35	35–<40	40–<45	45+	Total
39–44	%	40	6.6	11	11	13	32	31	14
	n	5	61	79	185	98	34	16	479
45–49	%	0	9.2	18	11	17	14	22	14
	n	12	65	125	246	143	58	23	672
50–54	%	0	7.9	17	15	23	34	25	18
	n	8	101	251	490	215	77	44	1,186
55–59	%	18	12	19	21	31	38	23	22
	n	11	126	348	568	179	80	35	1,347
60–64	%	6.3	17	25	22	27	33	33	24
	n	16	197	508	743	244	79	45	1,832
65–69	%	0	24	25	29	29	28	38	27
	n	13	245	694	827	272	69	29	2,149
70–74	%	13	31	37	29	43	36	53	34
	n	8	177	467	447	119	33	15	1,266
75–79	%	0	33	35	39	39	50	0	37
	n	1	117	325	220	66	14	0	743
80+	%	100	38	42	51	57	75	0	45
	n	1	72	139	87	23	4	0	326
Total	%	9.3	21	27	24	28	32	30	25
	n	75	1,161	2,936	3,813	1,359	448	207	10,000

^*^Prevalences (%) are for a combined category of participants potentially suitable for randomisation to trial as they had IFG (fasting plasma glucose ≥6.1–<7.0 mmol/mol) and/or non*–*diabetic hyperglycemia (NDH; HbA1c ≥6%–<6.5% (≥42 to <48 mmol/mol)). Each cell shows overall numbers of participants (n) screened in each age & BMI category (total n = 10,000), with prevalence (%) of categories potentially suitable for randomisation into trial for each cell. All data are based on the first baseline sample.

2,172 (21.7%) participants had non - diabetic hyperglycemia (HbA1c ≥6.0%–<6.5%; ≥42–<48 mmol/mol). They were significantly older, and had significantly different anthropometric data, compared to those with a normal HbA1c <6. 0% (<42 mmol/mol) (Table 1).

967 (9.7%) had impaired fasting glucose (IFG; ≥6.1–<7.0 mmol/L), and 2,651 (26.5%) had the broader definition of impaired fasting glucose (fasting plasma glucose ≥5.6–<7.0 mmol/L; Table 2). The two IFG subcategories (fasting plasma glucose ≥5.6–≤6.0 mmol/L, or ≥6.1–<7.0 mmol/l) did not differ significantly from each other in any clinical or anthropometric data (Table 2).

The prevalence of participants with non - diabetic hyperglycemia (NDH; HbA1c ≥6%–<6.5%, ≥42–<48 mmol/mol) and/or IFG (fasting plasma glucose ≥6.1–<7.0 mmol/L) by age and BMI category is summarised (Table 3). This shows a combined population (n = 2,515; 25.2%) potentially eligible for randomisation into NDPS intervention trials. The commonest age group to attend for screening was 65–69 years old (n = 2,149; 21.5%), the commonest BMI group was 30–35 kg/m^2^ (n = 3, 813; 38.1%), and 29% of this combined age and BMI defined category had either NDH or IFG (Table 3).

### Discordance between glycemic categories

The distribution of glycemic categories is shown in Table 4. Only 6,057 (60.6%) had an entirely normal combination of HbA1c <6.0% (<42 mmol/mol) and a fasting plasma glucose <5.6 mmol/L. Only 487 (4.9%) of this population had both NDH (HbA1c ≥ 6.0%–<6.5%; ≥42–<48 mmol/mol) and IFG (fasting plasma glucose ≥6.1–<7.0 mmol/L) on first screening (Table 4).

**Table 4:** Distribution of glycemic categories (based on initial baseline data) by HbA1c or fasting plasma glucose in 10,000 high risk participants screened for randomisation into the Norfolk Diabetes prevention Study (NDPS).

			HbA1c % (mmol/mol)		
			<6%	≥6%–<6.5%	≥6.5%
			(<42)	(≥42–<48)	(≥48)
			Normal	NDH	Type 2 diabetes
Fasting plasma glucose (mmol/L)	<5.6	Normal	6067 (60.6%)	990 (9.9%)	59 (0.6%)
	≥5.6–<6.1	IFG	968 (9.7%)	625 (6.3%)	91 (0.9%)
	≥6.1–6.9	IFG	306 (3.1%)	487 (4.9%)	174 (1.74%)
	≥7.0	Type 2 diabetes	11 (0.1%)	70 (0.7%)	162 (1.6%)

Please note these data classifications are based on the first of two baseline sample(s) for HbA1c and fasting plasma glucose collected in 10,000 participants.

For HbA1c categories, non - diabetic hyperglycemia (NDH) is defined as HbA1c ≥6.0%–<6.5% (≥42–<48 mmol/mol) and Type 2 diabetes as HbA1c >6.5% (≥48 mmol/mol). For fasting plasma glucose categories, impaired fasting glucose (IFG) is defined as fasting plasma glucose ≥5.6–<6.1 mmol/L or ≥6.1–<7.0 mmol/l, depending on IFG definitions^11,16,23^ and Type 2 diabetes is defined as a fasting plasma glucose ≥7.0 mmol/l. All data are based on first baseline sample in 10,000 participants.

There was substantial discordance between glycemic categories (Tables 4–6) as 1,274 of the 2,651 participants (48.1%) with impaired fasting glucose (fasting plasma glucose ≥5.6 to <7.0 mmol/L) had a normal HbA1c (<6.0%,; <42 mmol/mol), and 306 of 967 participants (31.6%) with impaired fasting glucose (fasting plasma glucose ≥6.1–<7.0 mmol/L) had a normal HbA1c (<6. 0%; <42 mmol/mol). In addition, 990 of the 2,172 participants (45.6%) with NDH (HbA1c ≥ 6.0%–<6.5%, >42–<48 mmol/mol) had a normal fasting glucose (<5.6 mmol/L).

**Table 5:** Short term (median 40 days) regression and progression in fasting glucose based glycemic categories between paired baseline data in 2,208 participants with an elevated initial baseline fasting plasma glucose or HbA1c.

	Repeat baseline sample category				
	Normal	IFG_1_^b^	IFG_2_^b^	Type 2 diabetes	Total
**Baseline first fasting plasma glucose category**					
Normal (<5.6 mmol/L)^a^	392 (69.6%)	139 (24.7%)	31 (5.5%)	1 (0.2%)	563
Impaired fasting glucose_1_ (IFG_1_)^b^	121 (27.3%)	186 (41.9%)	125 (28.2%)	12 (2.7%)	444
Impaired fasting glucose_2_ (IFG _2_)^b^	74 (7.8%)	269 (28.3%)	494 (52.0%)	113 (11.9%)	950
Type 2 diabetes^b^	3 (1.2%)	15 (6.0%)	80 (31.9%)	153 (61.0%)	251

^a^Some participants with normal fasting plasma glucose (<5.6 mmol/L) are included, as they had an elevated HbA1c (6%, ≥42 mmol/mol) on same baseline sample. Please note final total sample size is lower than in Tables 1–3 as repeat sample not undertaken in small number of IFG _2_, and a larger population with IFG_1_ as outlined in methods.

^b^IFG_1_ defined as fasting plasma glucose ≥5.6–<6.1 mmol/L, IFG_2_ defined as fasting plasma glucose ≥6.1–<7.0 mmol/L and Type 2 diabetes defined as fasting plasma glucose ≥7.0 mmol/l^11,16^.

Median time between paired baseline samples was 40 days (interquartile range 27–69 days).

Data shown as n and % for each row.

**Table 6:** Short term (median 40 days) regression and progression in HbA1c based glycemic categories between paired baseline data in 2,208 participants with an elevated initial baseline fasting plasma glucose or HbA1c.

	Repeat baseline sample category			
	Normal	Non diabetic hyperglycema	Type 2 diabetes	Total
**Baseline first HbA1c category**				
Normal (HbA1c 6%, <42 mmol/mol)^a^	268 (76.4%)	83 (23.6%)	0 (0%)	351
Non diabetic hyperglycemia (NDH)^b^	312 (21.3%)	1047 (71.6%)	104 (7.1%)	1463
Type 2 diabetes^b^	1 (0.3%)	82 (20.8%	311 (78.9%)	394

^a^Some participants with normal HbA1c (<6%, <42 mmol/mol) are included as also had an elevated fasting plasma glucose (≥6.1 mmol/L) on same sample. Please note final total sample size is lower than in Tables 1–3 as repeat sample not always undertaken as outlined in methods.

^b^NDH defined as HbA1c ≥6.0%–6.5 (≥42 to <48 mmol/mol) and Type 2 diabetes defined as HbA1c ≥6.5% (≥48 mmol/mol).

Median time between paired baseline samples was 40 days (interquartile range 27–69 days). Data shown as n and % for each row.

### Screen detected Type 2 diabetes

The prevalence of screen detected Type 2 diabetes is shown (Tables 1 and 2).

### Reproducibility of glycemic category at repeat confirmatory baseline sampling (Tables 5–8)

Of these 10,000 participants, 2,483 (24.8%) were eligible for a repeat fasting plasma glucose and HbA1c as a confirmatory baseline test prior to randomisation into an intervention trials.

**Table 7:** Mean fasting plasma glucose and HbA1c in participants with non - diabetic hyperglycemia ^a^ or impaired fasting glucose where glycemic category changed (progressed or regressed), or remained unchanged, on a repeat second baseline sample taken a median 40 days later^a^.

	**Regressed**	**Unchanged**	**Progressed**	**Overall p**
**Non diabetic hyperglycemia**				
n	312	1047	104	
Fasting plasma glucose (mmol/L)	5.6 (0.6)***	5.9 (0.6)	6.1 (0.7)***	0.0001
HbA1c % (mmol/mol)	42.8 (1.1)***	43.9 (1.4)	45.4 (1.4)***	0.0001
HbA1c (%)	6.1 (0.1)***	6.2 (1.4)	6.3 (1.4)***	
**Impaired fasting glucose**				
n	343	494	113	
Fasting plasma glucose (mmol/L)	6.3 (0.2)***	6.4 (0.2)	6.6 (0.2)***	0.0001
HbA1c % (mmol/mol)	42.0 (3.9)***	44.2 (4.3)	46.6 (4.4)***	0.0001
HbA1c (%)	6.10 (0.36)***	6.2 (4.3)	6.4 (4.4)***	

^**a**^Non diabetic hyperglycemia (NDH) on first sample defined as HbA1c ≥6% (≥42–<48 mmol/mol) and impaired fasting plasma glucose as ≥6.1 mmol/L–<7.0 mmol/l. The term regression means the second baseline sample was HbA1c <6% (<42 mmol/mol) or fasting plasma glucose <6.1 mmol/L on repeat baseline sample. The term progression means the second baseline sample was HbA1c ≥6.5% (≥48 mmol/mol) or fasting plasma glucose ≥7.0 mmol/L. Median 40 days (interquartile range 27–69 days) between paired baseline data. Please note total sample size (n = 950) for IFG is slightly lower than in Tables 1–3 as repeat sample not undertaken.

***p < 0.001 compared to unchanged group. Across group comparisons by one-way ANOVA model, and two-way comparison is based on an independent samples test. Data shown as mean (SD).

**Table 8:** Mean fasting plasma glucose and HbA1c in participants with a diabetes diagnostic range result on first baseline sample, who regressed to a non - diabetes diagnostic category, or remained unchanged, on the repeat second baseline sample taken a median 40 days later^a^.

	Regressed	Unchanged	Overall p
**Type 2 diabetes (based on HbA1c)**			
n	83	311	
Fasting plasma glucose (mmol/L)	6.2 (0.8)***	7.3 (1.8)	0.0001
HbA1c % (mmol/mol)	48.9 (1.54)***	54.7 (9.7)	0.0001
HbA1c (%)	6.6 (0.14)***	7.2 (0.89)	0.0001
**Type 2 diabetes (based on fasting plasma glucose)**			
n	98	153	
Fasting plasma glucose (mmol/L)	7.3 (0.5)***	8.5 (2.0)	0.0001
HbA1c (mmol/mol)	47.1 (6.5)***	56.7 (13.2)	0.0001
HbA1c (%)	6.5 (0.54)	7.3 (1.1)	0.0001

^**a**^Type 2 diabetes on first sample defined as an HbA1c ≥6.5% (≥48 mmol/mol) *and/or* fasting plasma glucose ≥7.0 mmol/L, and median time between paired sample 40 days (interquartile range 27–69 days) later. The term regression in this Table means the second baseline sample recorded was in a non - diabetes diagnostic category (HbA1c <6.5%, <48 mmol/mol or fasting plasma glucose <7.0 mmol/L).

***p < 0.001 compared to unchanged group.

Data shown as mean (SD).

After a median 40 days later (interquartile range 27–69 days) and prior to any programme intervention, repeat baseline confirmatory testing showed that 36.1% of 950 participants with IFG (fasting plasma glucose ≥6.1–<7.0 mmol/L) now recorded a fasting plasma glucose of <6.1 mmol/L (Table 5). In addition, 21.3% of 1,463 participants with NDH (HbA1c ≥ 6.0%–<6.5%, ≥42–<48 mmol/mol) now recorded a normal HbA1c (<6%, <42 mmol/mol) on repeat baseline testing (Table 6).

At the same baseline sampling interval, participants with newly detected Type 2 diabetes at first testing, 39% of 251 participants with Type 2 diabetes based on fasting plasma glucose ≥7.0 mmol/L, and 21.1% of 394 participants with Type 2 diabetes based on HbA1c 6.5% (≥48 mmol/L) now recorded a non - diabetes range repeat measurement (Tables 5 and 6).

A small proportion of participants with NDH (HbA1c ≥ 6%–<6.5%, ≥42–<48 mmol/mol) or IFG (fasting plasma glucose ≥6.1–<7.0 mmol/L) at first baseline measurement recorded a diabetes range repeat baseline measurement (7.1% and 11.9% respectively; Tables 5 and 6).

The biochemical differences between subgroups who apparently regressed, progressed, or remained in the same glycemic category between paired baseline samples are shown (Tables 7 and 8). There were highly significant differences in mean HbA1c and fasting plasma glucose at first baseline sampling between these categories, with the ‘regression’ groups having significantly lower mean plasma glucose and HbA1c than ‘progression’ group (Tables 7 and 8).

### Predictability of concordance in baseline results for an initially abnormal (42–47 mmol/mol) HbA1c

In the population with NDH, the probability of regression to a normal HbA1c result on repeat baseline testing was highly dependent on the initial HbA1c value (Table 9). For those with an initial HbA1c of 42 mmol/mol, 40.5% (95% CI 35.6–45.6) regressed to a normal baseline result, compared to 1.1% (95% CI 0–6.1) for those with an initial HbA1c of 47 mmol/mol. Participants with an NDH HbA1c of 45, 46, or 47 mmol/mol all had a >90% chance of having a concordant NDH HbA1c on repeat baseline testing. For those below this value (42, 43, or 44 mmol/mol), subcategorising by fasting plasma glucose data at baseline gave added value in predicting NDH HbA1c concordance. For example, although 40.5% (95% CI 35.6–45.6) of those an initial HbA1c of 42 mmol/mol regressed to normal on repeat baseline testing, this value was only 17% in those with an additionally elevated fasting plasma glucose (6.1–6.9 mmol/l), but 57% in those with an additionally normal fasting plasma glucose (4.5–4.9 mmol/l).

**Table 9:** Percentage of patients (% and 95% CI) with an initial non- diabetic hyperglycaemia (NDH) range HbA1c (42–47 mmol/mol) who then recorded a normal HbA1c (<42 mmol/mol) on a repeat baseline sample a median 40 days later, stratified by initial HbA1c.

Initial HbA1c (mmol/mol)	% normal (<42 mmol/mol) on repeat (95% CI)
42	40.5 (35.6–45.6)
43	29.4 (24.4–34.9)
44	13.7 (10.0–18.2)
45	6.6 (3.8–10.7)
46	6.1 (2.9–10.9)
47	1.1 (0.0–6.1)

NDH defined as HbA1c ≥6.0%–6.5% (≥42 to <48 mmol/mol) and median time between paired baseline samples was 40 days (interquartile range 27–69 days).

## Discussion

There are two principal findings relevant to normal clinical practice and clinical prevention programmes in these data.

Firstly, we found that the NDH population (HbA1c ≥ 6.0%–<6.5%; ≥42–<48 mmol/mol), were very heterogenous with a very high rate of discordance between high risk glycemic categories. Nearly half of the high risk NDH subjects (now the dominant high risk glycemic category in current clinical practice in many countries), had a completely normal fasting plasma glucose (<5.6 mmol/L) and would be at much lower risk of Type 2 diabetes than the smaller group with two combined high risk categories. This heterogeneity has been described in cross sectional and epidemiological studies, but not in the context of a mass population screening programme for risk stratification and entry to a very large diabetes prevention trial.

Secondly, we found that about one - quarter of the population with a biochemical high risk glycemic category (NDH or IFG) recorded a normal result on repeat baseline measurement a median 40 days later, making it difficult to give participants an accurate risk estimate and correct randomisation pathway. This reinforces the need for paired baseline concordant data before giving a diagnosis of NDH. For NDH participants with an HbA1c of 45–47 mmol/mol inclusive, there was a more than 90% likelihood of a repeat baseline result also showing NDH range HbA1c and this allows confidence in correct diagnosis and risk categorisation for these subjects. For NDH participants with HbA1c of 42–44 mmol/mol, there is a high rate of regression to normal on repeat baseline testing, and subcategorization by fasting plasma glucose data allows more accurate risk stratification and diagnosis. The groups that regressed to normal between baseline testing also had a significantly lower mean HbA1c and fasting plasma glucose, closer to the lower diagnostic threshold, than those that did not regress. These data suggest clinicians need to be more aware of the diagnostic imprecision of a single data point close to these thresholds, and the need to avoid a lifelong misdiagnosis.

These are novel observations in the context of stratifying for entry into a diabetes prevention trial or clinical prevention programme, and have significant implication for clinical practice.These data suggest the majority of people with NDH entering clinical prevention programmes are at much lower risk than assumed as they also have a normal fasting glucose, or regress to normal within days of testing. These data are relevant to primary care clinicians and planners, as an estimated 10.7% of the adult population in England now have NDH^23^ and UK national guidance is that all of these patients (perhaps 4 million people in England) should have targeted diet and lifestyle advice to reduce the risk of Type 2 diabetes^11^. It remains unclear who will deliver this and where clinical resources are limited it seem sensible to identify those at genuinely highest risk using paired baseline data and both fasting glucose and HbA1c who are most likely to benefit from an intervention. This approach to risk assessment is similar to that used in mild hypertension or dyslipidaemia, with the use of multiple variables and repeat measurements to determine intervention and possible benefit. This additional glucose categorisation also appears to have cardiovascular risk assessment benefits in this population^25^. This approach may also have value in capacity planning for other national prevention initiatives^16^.

The diagnostic cut point of HbA1c ≥6.5% (≥48 mmol/mol) for diabetes is supplanting glucose based criteria^11,17,18^. This policy shift generates a new large high risk population with HbA1c >6%–<6.5% (≥42–<48 mmol/mol)^11,18^, categorised as ‘high risk’ for type 2 diabetes and eligible for intensive lifestyle intervention^11^. There is little trial outcome data for diabetes prevention benefit in this large ‘high risk’ population with an isolated elevated HbA1c and a normal fasting plasma glucose, and yet this is the largest population entering the NHS England national prevention programme, although other national models use a range of glycemic and biometric entry points^16^. Treating this entire population^23^ as a homogenous group ignores the rich epidemiological data describing the added value of further risk stratification using both HbA1c and fasting glucose in predicting Type 2 diabetes incidence, and the discordance between the various ‘at risk’ categories. The sub categorisation of the ‘high risk’ NDH population with HbA1c ≥6.0%–<6.5% (≥42 to <48 mmol/mol) by fasting glucose identifies subgroups at very different absolute and relative risks of Type 2 diabetes^6,7,17^. The presence of a normal fasting plasma in the ‘high risk’ population with HbA1c ≥6.0%–<6.5% (≥42–<48 mmol/mol) identifies a subgroup at a lower annual risk of progression to Type 2 diabetes, at perhaps only 1–2.5%^19–22^, while the combination of a ‘high risk’ HbA1c ≥6%–<6.5% (≥42–<48 mmol/mol) and an elevated fasting plasma glucose identifies a subgroup at a much higher relative and absolute risk of incident type 2 diabetes^17–22^. This suggests a difference in progression rates of between perhaps 4.5% over 3 years for those with NDH and a normal fasting plasma glucose, and perhaps up to 24% for those with NDH *and* an elevated fasting plasma glucose^17–22^. As the absolute and relative risk of Type 2 diabetes in NDH can be so much better defined with an additional fasting glucose data, it seems clinically sensible to make a more accurate risk estimate by using additional fasting glucose data, and focussing on highest risk NDH groups, rather than a blanket application of an intervention dose in all subjects.

We found a high proportion (21.3%) of participants with NDH had a normal HbA1c result (<6%, <42 mmol/mol) on repeat baseline testing, and 36.1% with IFG (6.1–6.9 mmol/L) had a fasting plasma glucose <6.1 mmol/L after a median 40 days, and before any trial intervention. This very rapid short term regression is a relatively novel observation in a clinical prevention trial, and raises questions about the using a single glycemic data point for entry to prevention programmes, and how to interpret later clinical end points if many participants in fact had regressed to normal within weeks of first measurement. This observation may in part reflect self - intervention and lifestyle changes triggered by a first abnormal result^26^, or some regression to the mean. Groups that showed very short term regression or progression were characterised by a mean HbA1c or fasting plasma glucose closer to relevant diagnostic thresholds, and these changes may also reflect normal assay variance across this threshold. Regression of an abnormal glucose category to normal after very many years follow up (with or without an intervention), is well described^27–30^, but not very short term regression of an abnormal baseline HbA1c or fasting plasma glucose data to normal for subjects entering a prevention trial or programme, beforeintervention. This means that as well as one half of the NDH patients having a normal fasting glucose about one quarter also return to normal after a few days on repeat testing. It is not possible to assume that those who regress to normal are still at the same high risk, as persistently abnormal glucose categories have a higher predictive value for Type 2 diabetes than isolated measurements that regress^31,32^. However, the true risk of Type 2 diabetes or adverse cardiovascular outcomes experienced by this unusual population with discordant baseline results remains to be seen, and will be available with very long term follow up and accurate outcome data planned after the end of the programme in 2018. These data also show clearly the diagnostic value in the current policy of using paired concordant baseline data in diagnosing diabetes as only 78.9% of those with screen detected Type 2 diabetes (≥6.5%, ≥48 mmol/L) had a diabetes range HbA1c a median of 40 days later.

Biochemical measurements for diagnosis or monitoring such as HbA1c need to be interpreted with an understanding of Uncertainty of Measurement (UoM), which includes the Total Analytical Error (TAE) and Biological Variation. Components of TAE are the analytical imprecision and bias of the method, and Sigma-Metrics (SM) provides a benchmark on which a process can be characterised and incorporates both imprecision and bias and the SM targets for HbA1c have been published^33^. The methods used in NDPS conform to quality standards and methods standardised to the International Federation of Clinical Chemistry and Laboratory Medicine Reference measurement procedure, and the analytical imprecision has been shown to be <3% CV and within-individual biological variation is relatively small compared to the between-person variation^34,35^ in people without diabetes. Based on UoM, a change of >4 mmol/mol in measured HbA1c reflects a true change in glycemic category and a difference of 6.0% to 6.5% (42 to 48 mmol/mol) in a repeat measurement may simply be accounted for by UoM. This uncertainty needs to be acknowledged when categorizing participants, and reinforces the value of paired confirmatory data for glycemic categorization particularly for participants with results close to a diagnostic threshold. In addition, there are less commonly acknowledged contributions of lifestyle and genetic variance to glycation and HbA1c variability (independent of glycemic profiles)^36^, as are the effects of aging related impairment in insulin release and advanced glycation end products (AGE) on insulin sensitivity^37–39^.

One strength of this study is that these 10,000 subjects were drawn from a large ‘at risk of Type 2 diabetes’ population identified on routine primary care databases, using the simplest of database search terms (age, BMI and/or glycaemic data). This approach is easily translatable to most primary care systems, and is similar both to approaches used in many case finding prevention trials and to the NHS England national diabetes prevention programme. The population studied are also relatively homogenous in terms of age banding and ethnicity, and all biochemical and biometric measurements were undertaken in the same laboratory, and by a small number of trained observers. Although the 10,000 subjects described in this study are drawn from a larger approached sample, it is not possible to comment on any differences between the consented 10,000 and those who did not respond to initial contact. This population is a self - selecting sample to some degree therefore, although likely to be similar to the sort of participants who would self - select to participate in any clinical prevention programme or diet and lifestyle diabetes prevention programme, and has validity in terms of populations entering prevention programmes. The data in this paper can not necessarily be translated to other populations with different ethnicities or identified through other screening strategies, although the suggested approach to correct risk stratification should be applied to all populations. We are unable to be clearer on the mechanisms behind our novel observation of short term regression to normality in NDH after 40 days at baseline, and before any entry into the trial intervention. If this regression was apparent with only a few days between paired baseline measurements this would makes it more likely to be related to UoM in assay measurements, rather than participants making changes in lifestyle between paired baseline measurements, but the interval between paired results was a median 40 days. One further limitation of this study is that we have only paired baseline data for *entry* to trial, and not at the several interval testing time points during 40 month follow up, so we are not aware if this short term regression to normality in paired samples is also apparent in HbA1c and glucose data collected at interval (non - end point) tests – clearly if this was the case, it would have very major implications for accurate end point determination in both prevention trials and programmes.

These current data suggest very many people entering national prevention programmes or trials based solely on a single elevated HbA1c are in fact at much lower risk than is assumed. Risk categorization using both fasting plasma glucose and HbA1c data, the use of paired baseline data prior to entry into clinical or research programmes, and awareness of diagnostic imprecision would mitigate some of these difficulties, and avoid overestimation of risk and a lifelong misdiagnosis. This policy would also allow a more focussed risk estimation to identify those truly at highest risk of Type 2 diabetes, and most likely to benefit from intervention. These data add to the literature on the over diagnosis of risk (with associated stigma and increased anxiety) and overstatement of potential benefit, particularly in populations withisolated NDH where trial evidence of diabetes prevention benefit form lifestyle intervention is modest^14,15,39^.
